# Nursing diagnoses for people hospitalized with heart failure: an integrative review

**DOI:** 10.1590/0034-7167-2023-0471

**Published:** 2024-07-29

**Authors:** Ana Paula Dias de Oliveira, Lucas Garcia Lima, Vinicius Batista Santos, Larissa Maiara da Silva Alves Souza, Juliana de Lima Lopes, Alba Lucia Bottura Leite de Barros

**Affiliations:** I Universidade Federal de São Paulo. São Paulo, São Paulo, Brazil; II Sociedade Beneficente Israelita Hospital Albert Einstein. São Paulo, São Paulo, Brazil

**Keywords:** Nursing Diagnosis, Heart Failure, Review, Persons, Standardized Nursing Terminology, Diagnóstico de Enfermería, Insuficiencia Cardíaca, Revisión, Personas, Terminología Normalizada de Enfermería

## Abstract

**Objectives:**

to identify in the literature the main nursing diagnoses according to the NANDA-I diagnostic classification for people hospitalized with heart failure.

**Methods:**

an integrative literature review, carried out in February 2019 and updated in July 2023, in the MEDLINE via PubMed, LILACS, SciELO and CINAHL databases. Given the use of acronym PEO, studies without a time cut in Portuguese, English and Spanish were included. Descriptive analysis was carried out to present the identified information.

**Results:**

analysis of 27 articles identified 24 nursing diagnoses, with emphasis on Decreased Cardiac Output, Excessive Fluid Volume, Decreased Activity Tolerance and Fatigue.

**Final Considerations:**

evidence can contribute to better diagnostic decisions centered on people with heart failure in search of more assertive health results and have the potential to support future studies on a possible syndromic pattern in this population.

## RESUMEN

**Objetivos:**

identificar en la literatura los principales diagnósticos de enfermería según la clasificación diagnóstica NANDA-I para personas hospitalizadas con insuficiencia cardíaca.

**Métodos:**

revisión integrativa de la literatura, realizada en febrero de 2019 y actualizada en julio de 2023, en las bases de datos MEDLINE vía PubMed, LILACS, SciELO y CINAHL. Dado el uso del acrónimo PEO, se incluyeron estudios sin corte de tiempo en portugués, inglés y español. Se realizó un análisis descriptivo para presentar la información identificada.

**Resultados:**

el análisis de 27 artículos identificó 24 diagnósticos de enfermería, con énfasis en Disminución del Gasto Cardíaco, Volumen Excesivo de Líquidos, Disminución de la Tolerancia a la Actividad y Fatiga.

**Consideraciones Finales:**

la evidencia puede contribuir a mejores decisiones diagnósticas centradas en personas con insuficiencia cardíaca en busca de resultados de salud más asertivos y tiene el potencial de sustentar futuros estudios sobre un posible patrón sindrómico en esta población.

## INTRODUCTION

Heart failure (HF) is a health condition with high prevalence worldwide^(1)^. It exhibits significant social and economic burden, due to the impact on quality of life, increase in hospitalizations, number of deaths and hospital costs^(2)^. These facts highlight HF as a top public health priority^(3)^.

In Brazil, results of an analysis revealed significant hospitalization and mortality rates due to HF, which highlights the need to improve the results of care for this syndrome^(4)^.

HF is defined as any structural and/or functional impairment of blood volume ejection by the heart resulting in an intricate clinical syndrome with typical signs and symptoms^(5)^. Due to the complexity of HF, varied human responses may be affected as a consequence of the physiological mechanisms involved in the disease and its consequences. These people’s responses to health problems can be described by nurses using standardized languages^(6)^.

In this regard, a nursing diagnosis (ND) is used to describe the clinical understanding of human responses^(7)^ through critical thinking skills^(8)^, where nurses use NDs to plan nursing interventions (NIs) for achieving positive health outcomes^(9)^.

The most used terminology and considered the most researched ND vocabulary are NANDA-I taxonomy NDs^(10-11)^. Furthermore, it is the only one that presents well-defined criteria regarding the levels of accuracy of the constituent NDs in its conformation^(7)^.

NDs have been used to describe groups of people with specific health conditions, supporting decisions about clinical foci in different areas^(6)^. Furthermore, the identification of the most frequently identified NDs that occur together in specific health conditions can constitute a syndrome diagnosis. Syndrome NDs represent “a clinical judgment concerning a specific cluster of nursing diagnoses that occur together, and are best addressed together and through similar interventions”^(7)^. Thus, the present study aimed to identify the ND studied in people hospitalized with HF considering the need to compile and synthesize the main updated nursing research on ND in the context of hospitalization associated with HF and that can support future studies on a possible syndromic pattern in this population.

## OBJECTIVES

To identify NDs in the scientific literature according to the NANDA-I diagnostic classification for people hospitalized with HF.

## METHODS

### Ethical aspects

Due to the open access to the studies included in this review and because they do not contain documents with confidential data, assessment by a Research Ethics Committee was not necessary.

### Study design

This is an integrative literature review developed in six stages^(12)^ and based on Preferred Reporting Items for Systematic Review and Meta-Analyses (PRISMA) guidelines^(13)^.

### Study protocol

The six stages for carrying out this integrative review are described below:

1. Research question selection: what are the NDs identified for people hospitalized with HF evidenced in the literature?

The definition of the question included an adaptation of acronym PEO^(14)^, where P (population of interest) = people with HF; E (exposure of interest) = hospitalized; and O (outcome) = main NDs.

The search in databases was carried out in February 2019, with an update in July 2023. The studies were retrieved on the same date in order to eliminate bias. To select the articles, the Latin American and Caribbean Literature in Health Sciences (LILACS), MEDLINE via the PubMed portal, Scientific Electronic Library Online (SciELO) and Cumulative Index to Nursing and Allied Health Literature (CINAHL) databases were searched. The exploration of other databases was not expanded because the first search focused on selected databases. The tactics used to retrieve the articles were adjusted for each one, considering the focus of the question and the inclusion criteria of the integrative review.

The determination of controlled descriptors was referenced in the Descriptors in Health Sciences (DeCS), Medical Subject Headings (MESH) terms and CINAHL headings. The Boolean operators “*E*” and “*OU*” were used for the search in Portuguese, and “AND” and “OR” for the search in English. Chart 1 presents the search strategy adopted for the databases.

**Chart 1:** Search strategy used in databases, Brazil, 2023

Database	Strategy used
PubMed	heart failure AND (“nursing diagnosis” OR “nursing diagnoses” OR nursing diagnosis [mh])
CINAHL	heart failure AND (“nursing diagnosis” OR “nursing diagnoses”)
LILACS	(“heart failure” OR “*insuficiência cardíaca*”) AND (“*diagnóstico de enfermagem*” OR “nursing diagnosis” OR “nursing diagnoses”)
SciELO	(heart failure OR *insuficiência cardíaca*) AND (*diagnóstico de enfermagem* OR nursing diagnosis OR nursing diagnoses)

**Figure 1:**
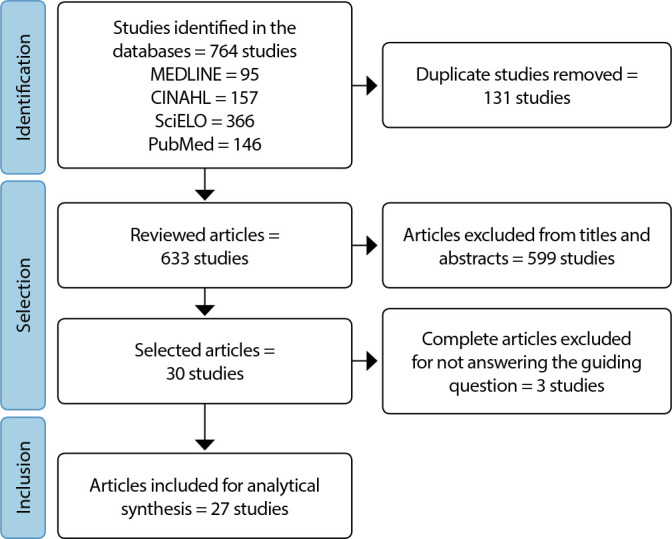
Flowchart of search and selection of analyzed articles, Brazil, 2023

2. Determination of study inclusion criteria and sample choice: studies that identified NDs in people aged 18 or over hospitalized with HF, with no publication time cut, in Portuguese, Spanish or English, were included. Articles related to people with HF in outpatient and home care, as well as those under 18 years of age, which addressed other nursing terminologies with the exception of NANDA-I, which were not associated with the topic of ND in people hospitalized with HF, and articles duplicates, were excluded. Likewise, studies that did not present a free and full version, textbooks, editorials, letters to the editor, conference abstracts and other gray literature studies were excluded.

3. Establishment of pre-selected and selected studies: the titles and abstracts of retrieved articles were read and chosen by two reviewers separately, postgraduate students, with disagreements being resolved by consensus. Those that met the study criteria were read in full. Finally, 30 studies were chosen for full consideration, of which 27 were part of the final sample, according to the representation in Figure 1, as recommended by the PRISMA guideline^(13)^.

4. Representation of selected studies: study data were extracted and presented in a spreadsheet containing the following elements, such as bibliographic data, methodological design, study objective, level of evidence (LoE) and identified NDs.

5. Critical analysis of the findings: the articles were categorized according to the purpose of each one, such as identified or validated NDs, accuracy of identified NDs, clinical indicators of identified NDs and nursing outcomes (NOs).

6. Review presentation: a chart was used to synthesize the data, which included the following aspects: authors; year of publication; country; study design; objective; LoE; Qualis classification of the journal; and identified NDs.

Study assessment regarding LoE was conducted according to the Oxford Center Evidence-Based Medicine criteria, which is based on the study design, categorized into different levels: 1A - systematic review; 1B - controlled and randomized clinical trial; 2A - systematic review of cohort studies; 2B - cohort study and randomized clinical trial of poorer quality; 2C - research results; 3A - systematic case-control study review; 3B - case study - control; 4 - case reports; and 5 - expert opinion and non-systematic review^(15)^.

Journal classification by the Coordination for the Improvement of Higher Education Personnel (CAPES - *Coordenação de Aperfeiçoamento de Pessoal de Nível Superior*) through Qualis measures the power of articles considering the quality of scientific journals that are classified into eight strata, in descending order of value: A1, A2, A3, A4, B1, B2, B3, B4 and C^(16)^. This classification was based on the 2017-2020 quadrennium classification system.

Data exploration was carried descriptively. Chart 2 presents the results synthesized, coded in chronological order

**Chart 2:** Characterization of selected studies, Brazil, 2023

Study	Author, year and country	Design	Objective	LoE	Qualis	ND studied
S1^(17)^	Assis CC e*t al*. (2007) Brazil	Quasi-experimental	Assess NO and NI for FA in people with HF.	2C	A4	FA
S2^(18)^	Martins QC *et al*. (2010) Brazil	Cross-sectional	Validate DCO DCs clinically and according to the model presented by Fehring.	2C	B4	DCO
S3^(19)^	Park H (2010) United States of America	Descriptive	Identify the main ND, NI, NO and links using standardized nursing terminologies for people with HF.	2C	A1	Deficient Knowledge, DCO, Risk for Injury and Ineffective Airway Clearance
S4^(20)^	Pereira JM *et al*. (2011) Brazil	Cross-sectional	Identify the prevalence of ND and DC in people with cardiovascular conditions and describe the association with sociodemographic and clinical variables.	2C	B1	Anxiety, Acute Pain, DCO and Disturbed Sensory Perception
S5^(21)^	Aliti GB *et al*. (2011) Brazil	Cross-sectional	Identify the clinical manifestations exhibited in decompensated HF with the aim of identifying priority NDs.	2C	A3	DCO and EFV
S6^(22)^	Silva RS *et al*. (2011) Brazil	Case study	Describe the application of the Nursing Process for a person with HF according to NANDA-I, NIC and NOC terminologies.	4	B1	EFV, DAT, Acute Pain and Impaired Gas Exchange
S7^(23)^	Scherb CA *et al*. (2011) United States of America	Multicenter comparison	Classify and contrast the ten nursing NDs, NI, NO according to NANDA-I, NIC and NOC for people with HF.	2C	B4	DAT, DCO, Deficient Knowledge and Risk for Falls
S8^(24)^	Martins QCS *et al*. (2011) Brazil	Cross-sectional	Clinically validate EFV DCs in people with decompensated HF.	2C	A2	EFV
S9^(25)^	Martins QC *et al*. (2012) Brazil	Cross-sectional	Present the conceptual and operational definitions of DCO DCs in HF.	2C	B1	DCO
S10^(26)^	Matos LN *et al*. (2012) Brazil	Cross-sectional	Identify the frequency and probability of DC predicting DCO for people with HF being analyzed for heart transplantation.	2C	A2	DCO
S11^(27)^	Pereira JM *et al*. (2015) Brazil	Descriptive	Verify the accuracy of nurses in identifying FA, DAT and DCO.	4	A4	FA, DAT and DCO
S12^(28)^	Park H *et al*. (2015) United States of America	Descriptive	Identify the main NDs with related factors and signs/symptoms using NANDA-I for people with HF.	2C	A1	Deficient Knowledge, DCO, Risk for Injury, Ineffective Airway Clearance, Risk for Infection, DAT, Acute Pain, Impaired Skin Integrity, and EFV
S13^(29)^	Souza V *et al*. (2015) Brazil	Cross-sectional	Assess the clinical application of the operational definitions for the DAT, DCO and EFV DCs.	2C	A1	DAT, DCO and EFV
S14^(30)^	Pereira JM *et al*. (2016) Brazil	Longitudinal	Identify FA, DAT and DCO in HF and examine the connection between DC and the existence of said ND.	2C	A2	FA, DAT and DCO
S15^(31)^	Galvão PC *et al*. (2016) Brazil	Cross-sectional	Identify the main NDs for people with decompensated HF.	2C	B1	DCO, DAT and Ineffective Breathing Pattern
S16^(32)^	Gonçalves LW et al. (2016) Brazil	Case study	Identify the cardinal diagnoses, NO, and NI for a person with HF through OPT clinical reasoning.	4	B1	DCO and Risk for Bleeding
S17^(33)^	Linhares JC *et al*. (2016) Brazil	Longitudinal and cohort	Verify the clinical application of NOC in people with decompensated HF and EFV.	2B	A3	EFV
S18^(34)^	Silva Alves Souza LM *et al*. (2017) Brazil	Cross-sectional	Analyze the connection between the ND identified for people with HF and the hemodynamic profiles displayed in the assessment.	2C	A1	Risk for Infection, Bathing Self-Care Deficit, Risk for DCO, Risk for Falls and EFV
S19^(35)^	Ernandes EM *et al*. (2019) Brazil	Cohort	Examine ND accuracy for people with a chance of clinical worsening during hospitalization for decompensated HF.	2B	A3	Ineffective Breathing Pattern, DCO, Ineffective Peripheral Tissue Perfusion, Risk for Ineffective Respiratory Function and EFV
S20^(36)^	Nascimento MNR *et al*. (2019) Brazil	Document analysis retrospective	Analyze aspects of nursing care for people with HF in an institution specializing in cardiology.	2C	B1	Risk for Infection, Ineffective Breathing Pattern, Self-Care Deficit, DCO, Impaired Bed Mobility, Anxiety, Impaired Skin Integrity, Imbalanced Nutrition, Ineffective Airway Clearance and EFV
S21^(37)^	Costa MB *et al*. (2019) Brazil	Cohort	Identify the most prevalent NANDA-I NDs and analyze the connection between NDs and other variables with death.	2C	B3	Anxiety, Sexual Dysfunction, DAT and FA
S22^(38)^	Santos VB *et al*. (2020) Brazil	Cross-sectional	Identify the prevalence of FA in people with HF and analyze DC accuracy.	2C	A1	FA
S23^(39)^	Trojahn MM *et al*. (2020) Brazil	Cohort	Examine the performance of B-type natriuretic peptide in the presence of EFV DC.	2B	A3	EFV
S24^(40)^	Lemos DMP *et al*. (2020) Brazil	Quantitative quasi-experimental	Assess the success of a discharge program based on NANDA-I, NIC and NOC taxonomies.	2C	A4	Ineffective Health Self-Management
S25^(41)^	Vianna TA *et al*. (2021) Brazil	Integrative review	Examine the main EFV DCs for people with HF.	5	B2	EFV
S26^(42)^	Padua BLR *et al*. (2022) Brazil	Exploratory and descriptive	Structure the terms recorded in medical records from to NANDA-I ND and NIC NI.	2C	A3	Risk for Infection, DCO and EFV
S27^(43)^	Ferreira JF *et al*. (2022) Brazil	Integrative review	Depict the most common NDs for people with HF.	5	B3	DCO, DAT, Anxiety, FA and EFV

*S - study; LoE - level of evidence; ND - nursing diagnosis(es); DC - defining characteristics; HF - heart failure; NI - nursing interventions; NO - nursing outcomes; NIC - Nursing Interventions Classification; NOC - Nursing Outcomes Classification; DCO - decreased cardiac output; EFV - Excessive Liquid Volume; DAT - Decreased Activity Tolerance; FA - Fatigue; OPT - Outcome-Present State-Test.*

## RESULTS

Initially, 764 articles were retrieved, of which 27 were included in the study, as shown in Figure 1.

Chart 2 presents a synthesis of the process of characterizing and classifying the quality of studies and journals of the 27 selected studies.

Most works were published in 2011 (n=5), followed by 2016 (n=4), 2015, 2019 and 2020 (n=3 each), 2010, 2012 and 2022 (n=2 each) and 2007, 2017 and 2021 (n=1 each). Of the 27 studies, 26 came from nursing journals and one from a medical journal.

Among those included, 12 were published in English and Portuguese, seven in Portuguese only, six in English, and two in English, Portuguese and Spanish. The studies were mostly carried out in Brazil (n=24; 88.9%), and the rest were carried out in the United States of America (n=3; 11.1%).

As for LoE, level 2C was the most frequent (n=19; 70.4%), followed by levels 2B and 4 (n=6; 22.2% each), and level 5 (n=2; 7, 4%). Most studies (n=16; 61.5%) came from journals with Qualis A.

As for the research design of selected articles, cross-sectional (n=10; 37.1%), descriptive (n=3; 11.1%), cohort (n=3, 11.1%), quasi-experimental (n=2; 7.4%), case (n=2; 7.4%), reviews (n=2; 7.4%), longitudinal (n=2; 7.4%), multicenter comparison (n=1; 3.7%), descriptive exploratory (n=1; 3.7%) and retrospective studies with document analysis (n=1; 3.7%) were found in the sample.

With regard to the object, most publications aimed to identify the most prevalent NDs (n=17; 63.0%), in addition to validating the NDs (n=2; 7.4%), identifying and/or analyzing the defining characteristics (DCs) of NDs (n=3; 11.1%), assessing NO after NI (n=3; 11.1%) and assessing the accuracy of studied NDs (n=2; 7.4%).

Moreover, 24 most frequent diagnostic labels in hospitalized people with HF were identified in the studies. The most cited ND per study can be seen in Chart 2.

Based on the results, the main ND studied in this review and their respective domains were compiled and ordered in decreasing order of frequency in Chart 3.

**Chart 3:** Distribution of nursing diagnoses studied in integrative review publications and their respective domains, São Paulo, São Paulo, Brazil, 2023

Nursing diagnosis	n	Domain
Decreased Cardiac Output	17	4. Activity/Rest
Excessive Liquid Volume	13	2. Nutrition
Activity Intolerance	9	4. Activity/Rest
Fatigue	6	4. Activity/Rest
Anxiety	4	9. Coping/Stress Tolerance
Risk for Infection	4	11. Safety/Protection
Deficient Knowledge	3	5. Perception/Cognition
Ineffective Airway Clearance	3	3. Elimination and Exchange
Acute Pain	3	12. Comfort
Ineffective Breathing Pattern	3	4. Activity/Rest
Risk for Injury	2	11. Safety/Protection
Imbalanced Nutrition: Less Than Body Requirements	2	2. Nutrition
Risk for Falls	2	11. Safety/Protection
Bathing Self-Care Deficit	2	4. Activity/Rest
Impaired Skin Integrity	2	11. Safety/Protection
Risk for Decreased Cardiac Output	1	4. Activity/Rest
Impaired Gas Exchange	1	3. Elimination and Exchange
Ineffective Health Self-Management	1	1. Health Promotion
Risk for Bleeding	1	11. Safety/Protection
Ineffective Peripheral Tissue Perfusion	1	4. Activity/Rest
Disturbed Sensory Perception	1	5. Perception/Cognition
Impaired Physical Mobility	1	4. Activity/Rest
Risk for Impaired Cardiovascular Function	1	4. Activity/Rest
Sexual Dysfunction	1	8. Sexuality

In this review, 24 NDs were most frequently identified. The most prevalent NDs in the studies listed were Decreased Cardiac Output (DCO), followed by Excessive Fluid Volume (EFV), Decreased Activity Tolerance (DAT) and Fatigue (FA).

## DISCUSSION

The results of this review made it possible to gather and synthesize studies on the most frequently identified NDs in people hospitalized with HF.

The most significant prevalence of studies in this review was carried out in Brazil, highlighting the national representation in the search for knowledge in the area of standardized language systems. Brazilian performance in this regard may reflect: inclusion of Nursing Process (NP) use in the profession regulation in 1986 and, later, with a specific resolution on the use of NP in 2009; efforts to disseminate NANDA-I terminology through conferences; adoption of NP in all nursing programs; educational initiatives, incorporating Distance Learning from the Nursing Diagnosis Update Program (PRONANDA - *Programa de Atualização em Diagnósticos de Enfermagem*); and interest of graduate programs that concentrate a significant percentage of theses and dissertations addressing ND^(7)^. In the present review, no European studies were found, reflecting the limiting factor in the use of various classification systems used on that continent^(44)^. This reinforces the need for international collaboration that must be undertaken as a strategy to achieve results that are understood and shared across countries and languages^(45)^.

Research published predominantly in journals with grade A, considered the best concept in production in the field of nursing, reflects the quality and impact of scientific production on the subject^(16)^. In relation to LoE, the studies were, for the most part, cross-sectional, emphasizing ND prevalence, and this finding reveals the urgency of studies with more robust quality of evidence^(46)^.

In relation to NDs, in agreement with the present study, DCO, EFV and DAT were the most prevalent in the integrative review that sought to verify the knowledge generated and disseminated in the world literature on NDs in people hospitalized with HF^(47)^. Another recent review that included 11 studies showed DCO, DAT, Anxiety, FA and EFV among the most frequently encountered NDs^(43)^. However, the authors of that review did not restrict people in the context of outpatient care, including studies based on the International Classification for Nursing Practice (ICNP) in addition to NANDA-I^(43)^.

Three of the four most prevalent NDs in this review belong to domain four (Activity/Rest) in NANDA-I taxonomy^(7)^, reflecting the most affected human responses during HF decompensation, where NDs that occur as a consequence of the pathophysiological process involved, which is cardiac pump deficit, predominate.

Recently, an integrative review that described the priority NDs in people with HF identified that DCO was the most prevalent^(43)^. Another current study identified DCs and contributing factors to this diagnosis, with emphasis on identifying four new related factors (hyperglycemic stress, prone position, left lateral position and sleep deprivation), adding evidence that supports the maintenance of this ND in NANDA-I terminology^(48)^.

DCO was considered the priority diagnosis in several studies^(30-32)^. Other international^(21,23,28)^ and national studies have identified DCO as one of the most prevalent diagnoses in people hospitalized with HF^(20,27,29,35,42)^.

In agreement with the investigation by Pereira (2016), this diagnosis was the most prevalent in an emergency unit, showing a frequency of 87.3% in the sample, and is correlated with decreased left ventricular stroke work index, altered heart rate, altered rhythm and altered contractility^(30)^.

Other research that aimed to identify the cardinal diagnoses, outcomes and NI for a person with HF using the Outcome-Present State-Test (OPT) clinical reasoning model described DCO as the central diagnosis, from which other diagnoses derive^(32)^.

In research that aimed to verify the existence and/or non-existence of FA, DAT and DCO in people with HF hospitalized in two centers located in Brazil, DCO was considered the priority in three weeks of follow-up, and DCs dyspnea, edema, jugular vein distension and decreased ejection fraction were identified as the most important^(30)^.

The main manifestations identified during hospital admission for DCO inference were dyspnea, paroxysmal nocturnal dyspnea, tiredness, edema, orthopnea and jugular vein distension^(21,36)^.

In people with HF being considered for heart transplantation, the DCs increased systemic vascular resistance, presence of S3 heart sound and decreased ejection fraction correlated with a reduction in cardiac index and DCO^(26)^.

Findings from another study demonstrated that DCO was established in 16% of participants. Even so, its diagnostic accuracy was considered high according to the Nursing Diagnosis Accuracy Scale version 2 with degrees of agreement between experts of 100%, validating that the high degree of importance, uniqueness and conformity of evidence indicate that DCO is specific and a priority for people with decompensated HF^(35)^. These findings reiterate that this ND requires the exercise of clinical reasoning for better diagnostic accuracy for its inference^(27)^.

In the search for greater accuracy, the conceptual and operational definitions for this diagnosis focusing on people with decompensated HF were described^(25)^. The DCO DCs were clinically validated in another research using the Fehring model in 29 people with decompensated HF, and it was identified that fatigue, dyspnea, edema, orthopnea, paroxysmal nocturnal dyspnea and increased central venous pressure constituted the main DCs, and weight gain, hepatomegaly, jugular vein distension, heart palpitations, pleural effusion, oliguria, coughing, clammy skin and altered skin color were secondary characteristics^(24)^.

Additionally, the clinical applicability of the definitions of DCO and EFV DCs was investigated, with five EFV DCs being significantly associated with the presence of DCO, such as positive hepatojugular reflex, altered mental status, altered respiratory pattern, decreased ejection fraction and ascites^(29)^.

Finally, research carried out through cross-mapping, using 115 medical records of people with decompensated HF in a hospital specializing in cardiology, mapped DCO along with other diagnoses and NIC NI, such as monitoring vital signs, water monitoring and positioning, among the most prevalent^(42)^.

EFV is frequently documented for people with congestive symptoms^(22,36,42-43)^, showing a relationship with the hemodynamic profiles displayed in clinical assessment^(34)^, in addition to high accuracy by nurses who evaluated people with HF in the first 24 hours of hospitalization^(35)^. The most common clinical manifestations are attributed to respiratory distress and right ventricular overload^(29)^ and are consistent with the study that identified dyspnea, orthopnea, edema, positive hepatojugular reflux, paroxysmal nocturnal dyspnea, pulmonary congestion and increased central venous pressure as the main DC in people with decompensated HF^(21)^. These same indicators or DCs in people with decompensated HF are correlated with an increase in biomarkers, such as type B natriuretic peptide and N-terminal pro-brain natriuretic peptide, which can be used as another parameter to improve diagnostic accuracy^(39)^. Furthermore, Nursing Outcomes Classification (NOC) NO for EFV also showed clinical applicability according to the increase in their score when comparing the means of the initial and final assessments in patients with decompensated HF^(33)^.

Despite requiring accuracy to support its accuracy^(27)^ and often presenting disagreement among experts^(30)^, DAT is also one of the most prevalent in studies that identify NDs in people with HF decompensation^(19,37,43)^.

This diagnosis was the most identified in a multicenter study that compared the ten most prevalent NDs in 302 electronic records^(23)^, being related to imbalance between oxygen supply and consumption^(31)^. Its main DCs are expresses fatigue, electrocardiogram change and abnormal heart rate response to activity^(29)^.

FA is an isolated ND from NANDA-I, and can also be a DC from DCO, DAT and seven other NDs linked to other domains and classes^(30)^. As a symptom, fatigue is a subjective and multifaceted phenomenon, affecting physical, psychological and social dimensions^(49)^.

Like ND, a recent integrative review identified FA as one of the most common^(43)^. Still, this ND has the potential to be misidentified in clinical practice, making it crucial to differentiate it from other NDs, especially with DAT, requiring reliable assessment methods for differentiation^(38)^. Furthermore, the need for self-report, associated with the subjectivity of the symptom, requires continuous and specific training to advance the diagnostic accuracy of this ND^(27)^.

Finally, despite the complexity that diagnostic accuracy imposes, FA constitutes a ND that demonstrates good evolution of the outcome indicators assessed after the institution of relevant NI^(17)^.

In addition to the high prevalence of DCO, EFV, DAT and FA, it was possible to glimpse a behavior that occurs together for people with decompensated HF. This behavior could characterize a syndromic diagnosis, given that they exhibit similar indicators and interventions.

### Study limitations

The search strategies adopted constitute an intrinsic limitation of integrative reviews due to the possibility of studies that were not included in the chosen tactic.

### Contributions to nursing

The update on the most frequently identified NDs for people hospitalized with HF contributes to the understanding of the main altered human responses in people with HF, directing the best diagnostic decision in search of more assertive health results and supported by a nursing practice model centered on people with HF. Finally, it can support future studies on a possible syndromic pattern in this population.

## FINAL CONSIDERATIONS

This study made it possible to identify that DCO, EFV, DAT and FA were the most frequently identified in people hospitalized with HF, adding evidence that this set of diagnoses can constitute a syndrome diagnosis.

Future investigations into the clustering of these most frequently identified NDs that occur together are needed to evaluate the existence of a NANDA-I syndrome ND in people hospitalized with HF.
