# Supplementary material for: Nursing diagnoses for people hospitalized with heart failure: an integrative review
Source: Rev Bras Enferm. 2024 Jul 29;77(3):e20230471. doi: 10.1590/0034-7167-2023-0471 (PMC11290743; doi:10.1590/0034-7167-2023-0471)
Supplement: Translation

# Translation: Diagnósticos de enfermagem para pessoas hospitalizadas com insuficiência cardíaca: revisão integrativa

**DOI:** 10.1590/0034-7167-2023-0471pt

**Authors:** Ana Paula Dias de Oliveira, Lucas Garcia Lima, Vinicius Batista Santos, Larissa Maiara da Silva Alves Souza, Juliana de Lima Lopes, Alba Lucia Bottura Leite de Barros

**Affiliations:** I Universidade Federal de São Paulo. São Paulo, São Paulo, Brasil; II Sociedade Beneficente Israelita Hospital Albert Einstein. São Paulo, São Paulo, Brasil

**Keywords:** Diagnóstico de Enfermagem, Insuficiência Cardíaca, Revisão, Pessoas, Terminologia Padronizada em Enfermagem

## Abstract

**Objetivos:**

identificar na literatura os principais diagnósticos de enfermagem de acordo com a classificação de diagnósticos da NANDA-I para pessoas hospitalizadas com insuficiência cardíaca.

**Métodos:**

revisão integrativa da literatura, realizada em fevereiro de 2019 e atualizada em julho de 2023, nas bases de dados MEDLINE via PubMed, LILACS, SciELO e CINAHL. Diante do uso do acrônimo PEO, incluíram-se estudos sem corte temporal de tempo em português, inglês e espanhol. Realizou-se análise descritiva para apresentar as informações identificadas.

**Resultados:**

análise de 27 artigos identificou 24 diagnósticos de enfermagem, com destaque para Débito Cardíaco Diminuído, Volume de Líquidos Excessivo, Tolerância à Atividade Diminuída e Fadiga.

**Considerações Finais:**

as evidências podem contribuir para a melhor decisão diagnóstica centrada na pessoa com insuficiência cardíaca em busca de resultados em saúde mais assertivos e têm potencial para subsidiar estudos futuros sobre um possível padrão sindrômico nessa população.

## INTRODUÇÃO

A insuficiência cardíaca (IC) é uma condição de saúde de alta prevalência em todo o mundo^(1)^_._ Exibe expressiva carga social e econômica em razão do impacto na qualidade de vida, aumento de internações, número de mortes e custos hospitalares^(2)^. Esses fatos destacam a IC como uma das principais prioridades de saúde pública^(3)^.

No Brasil, resultados de uma análise revelaram taxas de hospitalização e mortalidade por IC expressivas, o que expõe a necessidade de melhorar os resultados de atendimento dessa síndrome^(4)^.

A IC é definida como qualquer comprometimento estrutural e/ou funcional da ejeção do volume sanguíneo pelo coração, resultando em uma intricada síndrome clínica com sinais e sintomas típicos^(5)^. Em razão da complexidade da IC, respostas humanas variadas podem estar afetadas como consequência dos mecanismos fisiológicos implicados na doença e seus desdobramentos. Essas respostas das pessoas a problemas de saúde podem ser descritas pelos enfermeiros por meio de linguagens padronizadas^(6)^.

Nesse sentido, o diagnóstico de enfermagem (DE) é utilizado para descrever o entendimento clínico das respostas humanas^(7)^ por meio de habilidades de pensamento crítico^(8)^, onde os enfermeiros utilizam o DE para planejar as intervenções de enfermagem (IEs) para o alcance de resultados positivos em saúde^(9)^.

A terminologia mais utilizada e considerada o vocabulário de DE mais pesquisado são os de da taxonomia da NANDA-I^(10-11)^. Ademais, é a única que apresenta critérios bem delineados quanto aos níveis de precisão dos DEs constituintes em sua conformação^(7)^.

Os DEs têm sido usados para descrever grupos de pessoas com condições de saúde específicas, apoiando decisões sobre focos clínicos em diferentes áreas^(6)^. Além disso, a identificação dos DEs mais frequentemente identificados e que ocorrem juntos em condições de saúde específicas pode configurar um diagnóstico de síndrome. Os DEs de síndrome representam “*um julgamento clínico relativo a um determinado agrupamento de DE que ocorrem juntos, sendo mais bem tratados em conjunto e por meio de intervenções similares*”^(7)^. Nesse sentido, o presente estudo objetivou identificar os DEs estudados em pessoas hospitalizadas com IC, considerando a necessidade de compilar e sumarizar as principais pesquisas de enfermagem atualizadas sobre DE no contexto de internação associada à IC e que possam subsidiar estudos futuros sobre um possível padrão sindrômico nessa população.

## OBJETIVOS

Identificar na literatura científica os DEs de acordo com a classificação de diagnósticos da NANDA-I para pessoas hospitalizadas com IC.

## MÉTODOS

### Aspectos éticos

Em razão do acesso aberto aos estudos incluídos nesta revisão, e por não conterem documentos com dados confidenciais, não foi necessária a avaliação do Comitê de Ética em Pesquisa.

### Desenho do estudo

Trata-se de revisão integrativa da literatura desenvolvida em seis etapas^(12)^ e baseada nas diretrizes do *Preferred Reporting Itens for Systematic Review and Meta-Analyses* (PRISMA)^(13)^.

### Protocolo do estudo

As seis etapas para a realização desta revisão integrativa estão descritas a seguir:

1. Seleção da pergunta de pesquisa: quais são os DEs identificados para pessoas hospitalizadas com IC evidenciados na literatura?

A definição da pergunta contemplou uma adequação do acrônimo PEO^(14)^, onde P (população de interesse) = pessoas com IC; E (exposição de interesse) = hospitalizadas; e O (desfecho/*outcome*) = principais DEs.

A busca nas bases de dados foi realizada em fevereiro de 2019, com atualização em julho de 2023. Os estudos foram recuperados na mesma data de modo a dirimir viés. Para a seleção dos artigos, foram pesquisadas as bases de dados Literatura Latino-Americana e do Caribe em Ciências da Saúde (LILACS), MEDLINE via portal PubMed, biblioteca *online Scientific Electronic Library Online* (SciELO) e *Cumulative Index to Nursing and Allied Health Literature* (CINAHL). A exploração de outras bases de dados não foi ampliada em razão da primeira busca ter focado nas bases de dados selecionadas. As táticas utilizadas para recuperar os artigos foram ajustadas para cada uma, considerando o centro da pergunta e os critérios de inclusão da revisão integrativa.

A determinação de descritores controlados foi referenciada nos Descritores em Ciências da Saúde (DeCS), nos termos da *Medical Subject Headings* (MESH) e nos CINAHL *headings*. Os operadores booleanos “E” e “OU” foram utilizados para a busca em português, e “*AND*” e “*OR*”, na busca em inglês. A estratégia de busca adotada para as bases de dados foi apresentada no Quadro 1.

**Quadro 1:** Estratégia de busca utilizada nas bases de dados, Brasil, 2023

Bases de dados	Estratégia utilizada
PubMed	*heart failure AND* (“*nursing diagnosis*” *OR* “*nursing diagnoses*” *OR nursing diagnosis* [mh])
CINAHL	*heart failure AND* (“*nursing diagnosis*” *OR* “*nursing diagnoses*”)
LILACS	(“*heart failure*” *OR* “insuficiência cardíaca”) *AND* (“diagnóstico de enfermagem” *OR* “*nursing diagnosis*” *OR* “*nursing diagnoses*”)
SciELO	(*heart failure OR* insuficiência cardíaca) *AND* (diagnóstico de enfermagem *OR nursing diagnosis OR nursing diagnoses*)

2. Determinação dos critérios de inclusão de estudos e escolha da amostra: foram incluídos estudos que identificassem os DEs em pessoas iguais ou maiores de 18 anos hospitalizadas com IC, sem corte temporal de publicação, em português, espanhol ou inglês. Foram excluídos artigos relacionados a pessoas com IC em cuidado ambulatorial e domiciliar, assim como menores de 18 anos, que abordaram outras terminologias de enfermagem à exceção da NANDA-I, que não apresentaram associação com o tema sobre DE em pessoas hospitalizadas com IC e artigos duplicados. Do mesmo modo, foram excluídos estudos que não apresentassem versão livre e na íntegra, livros texto, editoriais, cartas ao editor, resumos de conferências e demais produções da literatura cinzenta.

3. Estabelecimento dos estudos pré-selecionados e selecionados: os títulos e resumos dos artigos resgatados foram lidos e elegidos por dois revisores separadamente, estudantes de pós-graduação, sendo as discordâncias resolvidas em consenso. Os que atenderam aos critérios do estudo foram lidos na íntegra. Por fim, 30 estudos foram escolhidos para apreciação na íntegra, dos quais 27 integraram a amostra final, de acordo com a representação na Figura 1, conforme recomendado pela diretriz PRISMA^(13)^.

**Figura 1:** Fluxograma da busca e seleção de artigos analisados, Brasil, 2023

4. Representação dos estudos selecionados: os dados dos estudos foram extraídos e apresentados em uma planilha contendo os seguintes elementos, como dados bibliográficos, desenho metodológico, objetivo do estudo, nível de evidência (NE) e DEs identificados.

5. Análise crítica dos achados: os artigos foram categorizados segundo a finalidade de cada um, como DEs identificados ou validados, acurácia dos DEs identificados, indicadores clínicos dos DEs identificados e resultados de enfermagem (REs).

6. Apresentação da revisão: foi utilizado um quadro com a síntese dos dados, que contemplou os seguintes aspectos: autores; ano da publicação; país; delineamento do estudo; objetivo; NE; classificação Qualis do periódico; e DEs identificados.

A avaliação dos estudos quanto ao NE foi conduzida segundo os critérios da *Oxford Centre Evidence-Based Medicine*, que é baseada no desenho do estudo, categorizados em níveis distintos: 1A - revisão sistemática; 1B - ensaio clínico controlado e randomizado; 2A - revisão sistemática de estudos de coorte; 2B - estudo de coorte e ensaio clínico randomizado de qualidade mais fraca; 2C - resultados de pesquisas; 3A - revisão sistemática de estudo de caso - controle; 3B - estudo de caso - controle; 4 - relatos de casos; e 5 - opinião de peritos e revisão não sistemática^(15)^.

A classificação de periódicos pela Coordenação de Aperfeiçoamento de Pessoal de Nível Superior (CAPES) por meio do Qualis mede o poder dos artigos considerando a qualidade dos periódicos científicos que são enquadrados em oito estratos, em ordem decrescente de valor: A1, A2, A3, A4, B1, B2, B3, B4 e C^(16)^. Essa classificação foi baseada no sistema de classificação do quadriênio de 2017-2020.

A exploração dos dados foi efetuada de forma descritiva. Os resultados foram sumarizados, codificados em ordem cronológica e apresentados no Quadro 2.

**Quadro 2:** Caracterização dos estudos selecionados, Brasil, 2023

Estudo	Autor, ano e país	Delineamento	Objetivo	NE	Qualis	DE estudado
E1^(17)^	Assis CC e*t al*. (2007) Brasil	Quase-experimental	Avaliar os RE e IE para o DE FA em pessoas com IC.	2C	A4	FA
E2^(18)^	Martins QC *et al*. (2010) Brasil	Transversal	Validar de modo clínico e de acordo com o modelo apresentado por Fehring as CDs do DE DCD.	2C	B4	DCD
E3^(19)^	Park H (2010) Estados Unidos da América	Descritivo	Identificar os principais DEs, IE, REs e vínculos usando terminologias de enfermagem padronizadas para pessoas com IC.	2C	A1	Déficit de Conhecimento, DCD, Risco de Lesão e Desobstrução Ineficaz das Vias Aéreas
E4^(20)^	Pereira JM *et al*. (2011) Brasil	Transversal	Identificar a prevalência dos DEs e as CDs de pessoas com condições cardiovasculares e descrever a associação com variáveis sociodemográficas e clínicas.	2C	B1	Ansiedade, Dor Aguda, DCD e Percepção Sensorial Perturbada
E5^(21)^	Aliti GB *et al*. (2011) Brasil	Transversal	Identificar as manifestações clínicas exibidas na IC descompensada objetivando identificar os DEs prioritários.	2C	A3	DCD e VLE
E6^(22)^	Silva RS *et al*. (2011) Brasil	Estudo de caso	Descrever a aplicação do Processo de Enfermagem para uma pessoa com IC de acordo com as terminologias da NANDA-I, NIC e NOC.	4	B1	VLE, TAD, Dor Aguda e Troca de Gases Prejudicada
E7^(23)^	Scherb CA *et al*. (2011) Estados Unidos da América	Comparativo multicêntrico	Classificar e contrastar os dez DE, IE, RE de enfermagem conforme a NANDA-I, NIC e NOC para pessoas com IC.	2C	B4	TAD, DCD, Conhecimento Deficiente e Risco de Quedas
E8^(24)^	Martins QCS *et al*. (2011) Brasil	Transversal	Validar, de modo clínico, as CDs do DE VLE em pessoas com IC descompensada.	2C	A2	VLE
E9^(25)^	Martins QC *et al*. (2012) Brasil	Transversal	Apresentar as definições conceituais e operacionais das CDs do DE DCD na IC.	2C	B1	DCD
E10^(26)^	Matos LN *et al*. (2012) Brasil	Transversal	Identificar a frequência e probabilidade das CDs predizerem DCD para pessoas com IC em análise para transplante cardíaco.	2C	A2	DCD
E11^27^	Pereira JM *et al*. (2015) Brasil	Descritivo	Verificar a acurácia dos enfermeiros na identificação dos DEs FA, TAD e DCD.	4	A4	FA, TAD e DCD
E12^(28)^	Park H *et al*. (2015) Estados Unidos da América	Descritivo	Identificar os principais DEs com fatores relacionados e sinais/sintomas utilizando a NANDA-I para pessoas com IC.	2C	A1	Déficit de Conhecimento, DCD, Risco de Lesão, Desobstrução Ineficaz das Vias Aéreas, Risco de Infecção, TAD, Dor Aguda, Integridade da Pele Prejudicada e VLE
E13^(29)^	Souza V *et al*. (2015) Brasil	Transversal	Avaliar a aplicação clínica das definições operacionais para as CDs dos DEs TAD, DCD e VLE.	2C	A1	TAD, DCD e VLE
E14^(30)^	Pereira JM *et al*. (2016) Brasil	Longitudinal	Identificar os DEs FA, TAD e DCD na IC e examinar a ligação entre as CDs com a existência dos referidos DEs.	2C	A2	FA, TAD e DCD
E15^(31)^	Galvão PC *et al*. (2016) Brasil	Transversal	Identificar os principais DEs para pessoas com IC descompensada.	2C	B1	DCD, TAD e Padrão Respiratório Ineficaz
E16^(32)^	Gonçalves LW et al. (2016) Brasil	Estudo de caso	Identificar os diagnósticos, REs e IEs cardinais para uma pessoa com IC por meio do raciocínio clínico OPT.	4	B1	DCD e Risco de Sangramento
E17^(33)^	Linhares JC *et al*. (2016) Brasil	Longitudinal e coorte	Verificar a aplicação clínica da NOC em pessoas com IC descompensada e o DE VLE.	2B	A3	VLE
E18^(34)^	Silva Alves Souza LM *et al*. (2017) Brasil	Transversal	Analisar a ligação dos DEs identificados para pessoas com IC com os perfis hemodinâmicos exibidos na avaliação.	2C	A1	Risco de Infecção, Déficit no Autocuidado no Banho, Risco de DCD, Risco de Quedas e VLE
E19^(35)^	Ernandes EM *et al*. (2019) Brasil	Coorte	Examinar acurácia diagnóstica de enfermagem para pessoas com chance de agravamento clínico durante hospitalização por IC descompensada.	2B	A3	Padrão Respiratório Ineficaz, DCD, Perfusão Tissular Cardiopulmonar Ineficaz, Risco de Função Respiratória Ineficaz e VLE
E20^(36)^	Nascimento MNR *et al*. (2019) Brasil	Retrospectivo de análise documental	Analisar os aspectos da assistência de enfermagem para pessoas com IC em instituição especializada em cardiologia.	2C	B1	Risco de Infecção, Padrão Respiratório Ineficaz, Déficit no Autocuidado, DCD, Mobilidade no Leito Prejudicada, Ansiedade, Integridade da Pele Prejudicada, Nutrição Desequilibrada, Desobstrução Ineficaz de Vias Aéreas e VLE
E21^(37)^	Costa MB *et al*. (2019) Brasil	Coorte	Identificar os DEs da NANDA-I mais prevalentes e analisar a ligação dos DEs e outras variáveis com o óbito.	2C	B3	Ansiedade, Disfunção Sexual, TAD e FA
E22^(38)^	Santos VB *et al*. (2020) Brasil	Transversal	Identificar a prevalência do DE FA em pessoas com IC e analisar a acurácia das CDs.	2C	A1	FA
E23^(39)^	Trojahn MM *et al*. (2020) Brasil	Coorte	Examinar o desempenho do peptídeo natriurético tipo B na vigência de CD de VLE.	2B	A3	VLE
E24^(40)^	Lemos DMP *et al*. (2020) Brasil	Quantitativo quase-experimental	Avaliar o êxito de um programa de alta baseado nas taxonomias NANDA-I, NIC e NOC.	2C	A4	Controle Ineficaz da Saúde
E25^(41)^	Vianna TA *et al*. (2021) Brasil	Revisão integrativa	Examinar as principais CDs do DE VLE para pessoas com IC.	5	B2	VLE
E26^(42)^	Padua BLR *et al*. (2022) Brasil	Exploratório e descritivo	Estruturar os termos registrados em prontuários para DEs da NANDA-I e IE da NIC.	2C	A3	Risco de Infecção, DCD e VLE
E27^(43)^	Ferreira JF *et al*. (2022) Brasil	Revisão integrativa	Retratar os DEs mais frequentes para pessoas com IC.	5	B3	DCD, TAD, Ansiedade, FA e VLE

*E - estudo; NE - nível de evidência; DE - diagnóstico(s) de enfermagem; CD - características definidoras; IC - insuficiência cardíaca; IEs - intervenções de enfermagem; REs - resultados de enfermagem; NIC - Nursing Interventions Classification; NOC - Nursing Outcomes Classification; DCD - Débito Cardíaco Diminuído; VLE - Volume de Líquido Excessivo; TAD - Tolerância à Atividade Diminuída; FA - Fadiga; OPT - Outcome-Present State-Test.*

## RESULTADOS

Foram recuperados, inicialmente, 764 artigos, dos quais 27 foram incluídos no estudo, conforme a Figura 1.

O Quadro 2 apresenta a síntese do processo de caracterização e classificação da qualidade dos estudos e dos periódicos dos 27 estudos selecionados.

A maioria dos trabalhos foi publicada em 2011 (n=5), seguidos de 2016 (n=4), 2015, 2019 e 2020 (n=3 cada), 2010, 2012 e 2022 (n=2 cada) e 2007, 2017 e 2021 (n=1 cada). Dos 27 estudos, 26 foram provenientes de periódicos da área de enfermagem e uma de revista médica.

Entre os incluídos, 12 foram publicados em inglês e português, sete, apenas em português, seis, somente em inglês, e dois, em inglês, português e espanhol. As publicações foram majoritariamente realizadas no Brasil (n=24; 88,9%), e as demais foram realizadas nos Estados Unidos da América (n=3; 11,1%).

Quanto ao NE, o nível 2C foi o mais frequente (n=19; 70,4%), seguido dos níveis 2B e 4 (n=6; 22,2% cada), e nível 5 (n=2; 7,4%). A maioria das publicações (n=16; 61,5%) foi proveniente de revistas com Qualis A.

Em relação ao delineamento de pesquisa dos artigos selecionados, evidenciaram-se na amostra estudos transversais (n=10; 37,1%), descritivos (n=3; 11,1%), coortes (n=3, 11,1%), quase-experimentais (n=2; 7,4%), de caso (n=2; 7,4%), revisões (n=2; 7,4%), longitudinais (n=2; 7,4%), comparativos multicêntricos (n=1; 3,7%), exploratórios descritivos (n=1; 3,7%) e retrospectivos com análise documental (n=1; 3,7%).

No que diz respeito ao objeto, a maioria das publicações visou identificar os DEs mais prevalentes (n=17; 63,0%), além de validar os DEs (n=2; 7,4%), identificar e/ou analisar as características definidoras (CDs) dos DEs (n=3; 11,1%), avaliar os REs após IEs (n=3; 11,1%) e avaliar a acurácia dos DEs estudados (n=2; 7,4%).

Foram apontados nos estudos 24 rótulos diagnósticos mais frequentes em pessoas com IC hospitalizadas. Os DEs mais citados por estudo podem ser visualizados no Quadro 2.

A partir dos resultados, os principais DEs estudados nesta revisão e seus respectivos domínios foram compilados e ordenados por ordem decrescente de frequência no Quadro 3.

**Quadro 3:** Distribuição dos diagnósticos de enfermagem estudados nas publicações da revisão integrativa e seus respectivos domínios, São Paulo, São Paulo, Brasil, 2023

Diagnóstico de enfermagem	n	Domínio
Débito Cardíaco Diminuído	17	4. Atividade/Repouso
Volume de Líquido Excessivo	13	2. Nutrição
Intolerância à Atividade	9	4. Atividade/Repouso
Fadiga	6	4. Atividade/Repouso
Ansiedade	4	9. Enfrentamento/Tolerância ao Estresse
Risco de Infecção	4	11. Segurança/Proteção
Conhecimento Deficiente	3	5. Percepção/Cognição
Desobstrução Ineficaz das Vias Aéreas	3	3. Eliminação e Troca
Dor Aguda	3	12. Conforto
Padrão Respiratório Ineficaz	3	4. Atividade/Repouso
Risco de Lesão	2	11. Segurança/Proteção
Nutrição Desiquilibrada: Menor Que As Necessidades Corporais	2	2. Nutrição
Risco de Queda	2	11. Segurança/Proteção
Déficit no Autocuidado no Banho	2	4. Atividade/Repouso
Integridade da Pele Prejudicada	2	11. Segurança/Proteção
Risco de Débito Cardíaco Diminuído	1	4. Atividade/Repouso
Troca de Gases Prejudicada	1	3. Eliminação e Troca
Controle Ineficaz da Saúde	1	1. Promoção da Saúde
Risco de Sangramento	1	11. Segurança/Proteção
Perfusão Tissular Cardiopulmonar Ineficaz	1	4. Atividade/Repouso
Percepção Sensorial Alterada	1	5. Percepção/Cognição
Mobilidade Física Prejudicada	1	4. Atividade/Repouso
Risco de Função Cardiovascular Prejudicada	1	4. Atividade/Repouso
Disfunção Sexual	1	8. Sexualidade

Nesta revisão, 24 DEs foram identificados mais frequentemente. Os DEs mais prevalentes nos estudos elencados foram Débito Cardíaco Diminuído (DCD), seguidos de Volume de Líquidos Excessivo (VLE), Tolerância à Atividade Diminuída (TAD) e Fadiga (FA).

## DISCUSSÃO

Os resultados desta revisão possibilitaram reunir e sintetizar os estudos sobre os DEs mais frequentemente identificados em pessoas hospitalizadas com IC.

A prevalência mais expressiva de estudos desta revisão foi nos realizados no Brasil, ressaltando a representatividade nacional na busca de conhecimento na área dos sistemas de linguagem padronizada. O desempenho brasileiro nesse tocante pode espelhar: a inclusão do uso do Processo de Enfermagem (PE) na regulamentação da profissão em 1986 e, posteriormente, com uma resolução específica sobre o uso do PE em 2009; os esforços na disseminação da terminologia da NANDA-I, por meio de conferências; a adoção do PE em todos os programas de enfermagem; as iniciativas educativas, incorporando Ensino a Distância do Programa de Atualização em Diagnósticos de Enfermagem (PRONANDA); e o interesse dos programas de pós-graduação que concentram porcentagem expressiva de teses e dissertações abordando DE^(7)^. Na presente revisão, não foram encontrados estudos europeus, refletindo o fator limitante do uso de vários sistemas de classificação utilizados naquele continente^(44)^. Isso reforça a necessidade de colaboração internacional que deve ser empreendida como estratégia para alcançar resultados compreendidos e compartilhados entre países e idiomas^(45)^.

As pesquisas publicadas predominantemente em revistas de conceito A, considerado o melhor conceito em produção no âmbito da enfermagem, refletem a qualidade e o impacto da produção científica sobre o assunto^(16)^. Em relação ao NE, os estudos foram, em sua maioria, do tipo transversal, enfatizando a prevalência dos DEs, e esse achado revela a premência de estudos com qualidade de evidência mais robusta^(46)^.

Em relação aos DEs em concordância com o presente estudo, DCD, VLE e TAD foram os mais prevalentes na revisão integrativa que buscou verificar o conhecimento gerado e veiculado na literatura mundial sobre DEs em pessoas hospitalizadas com IC^(47)^. Outra revisão recente que incluiu 11 estudos mostrou DCD, TAD, Ansiedade, FA e VLE entre os DEs mais encontrados^(43)^. Contudo, os autores da referida revisão não restringiram pessoas em contexto de cuidado ambulatorial, incluindo estudos baseados na Classificação Internacional para a Prática de Enfermagem (CIPE) além da NANDA-I^(43)^.

Três dos quatro DE mais prevalentes nesta revisão pertencem ao domínio quatro (Atividade/Repouso) na taxonomia da NANDA-I^(7)^, refletindo as respostas humanas mais afetadas na vigência de descompensação da IC, onde predominam os DEs que ocorrem como consequência do processo fisiopatológico envolvido, que é o déficit da bomba cardíaca.

Recentemente, revisão integrativa que descreveu os DEs prioritários em pessoas com IC identificou que DCD foi o mais prevalente^(43)^. Outro estudo atual identificou as CDs e os fatores contribuintes para esse diagnóstico com relevo para a identificação de quatro novos fatores relacionados (estresse hiperglicêmico, posição prona, posição lateral esquerda e privação do sono), somando indícios que sustentam a manutenção desse DE na terminologia da NANDA-I^(48)^.

O DE DCD foi considerado o diagnóstico prioritário em várias pesquisas^(30-32)^. Outros estudos internacionais^(21,23,28)^ e nacionais identificaram o DE DCD como um dos diagnósticos mais prevalentes em pessoas hospitalizadas com IC^(20,27,29,35,42)^.

Em concordância com a investigação de Pereira (2016), esse diagnóstico foi o mais prevalente em uma unidade de emergência, exibindo frequência de 87,3% na amostra, e está correlacionado com diminuição da fração de ejeção do ventrículo esquerdo, frequência cardíaca alterada, ritmo alterado e contratilidade alterada^(30)^.

Outra pesquisa que visou identificar os diagnósticos, resultados e IE cardinais para uma pessoa com IC utilizando o modelo de raciocínio clínico *Outcome-Present State-Test* (OPT) descreveu DCD como o diagnóstico central, do qual outros diagnósticos derivam^(32)^.

Em pesquisa que objetivou verificar a existência e/ou inexistência dos DEs FA, TAD e DCD em pessoas com IC hospitalizadas em dois centros localizados no Brasil, o DE DCD foi considerado o prioritário em três semanas de acompanhamento, e as CDs dispneia, edema, distensão da veia jugular e fração de ejeção diminuída foram identificadas como as mais importantes^(30)^.

As principais manifestações identificadas na vigência da admissão hospitalar para a inferência de DCD foram dispneia, dispneia paroxística noturna, cansaço, edema, ortopneia e distensão da veia jugular^(21,36)^.

Em pessoas com IC em análise para transplante cardíaco, as CDs resistência vascular sistêmica aumentada, terceira bulha e fração de ejeção diminuída correlacionaram-se com a redução do índice cardíaco e o DE DCD^(26)^.

Achados de outro estudo demonstraram que o DE DCD foi estabelecido em 16% dos participantes. Ainda assim, sua acurácia diagnóstica foi considerada alta de acordo com a Escala de Acurácia de Diagnósticos de Enfermagem, versão 2, com graus de concordância entre os peritos de 100%, validando que o elevado grau de importância, a singularidade e a conformidade dos indícios apontam que DCD é específico e prioritário para pessoas com IC descompensada^(35)^. Esses achados reiteram que esse DE exige o exercício do raciocínio clínico para uma melhor acurácia diagnóstica para sua inferência^(27)^.

Na busca de maior exatidão, as definições conceituais e operacionais para esse diagnóstico com foco em pessoas com IC descompensada foram descritas^(25)^. As CDs do DE DCD foram validadas clinicamente em outra pesquisa utilizando o modelo de Fehring em 29 pessoas com IC descompensada, sendo identificado que fadiga, dispneia, edema, ortopneia, dispneia paroxística noturna e pressão venosa central elevada constituíram as CDs principais, e ganho de peso, hepatomegalia, distensão da veia jugular, palpitações, crepitações, oliguria, tosse, pele úmida e alterações da cor da pele foram características secundárias^(24)^.

Adicionalmente, a aplicabilidade clínica das definições das CDs de DCD e VLE foi investigada, sendo que cinco CDs de VLE foram associadas de modo significante com a presença de DCD, como reflexo hepatojugular positivo, mudança no estado mental, mudança no padrão respiratório, fração de ejeção diminuída e ascite^(29)^.

Finalmente, pesquisa realizada por meio do mapeamento cruzado, utilizando 115 prontuários de pessoas com IC descompensada em hospital especializado em cardiologia, mapeou DCD junto de outros diagnósticos e as IEs da NIC, como monitoração de sinais vitais, monitoração hídrica e posicionamento, entre as mais prevalentes^(42)^.

O DE VLE é frequentemente documentado para pessoas com sintomas congestivos^(22,36,42-43)^, apresentando relação com os perfis hemodinâmicos exibidos na avaliação clínica^(34)^, além de alta acurácia por enfermeiros que avaliaram pessoas com IC nas primeiras 24 horas de internação^(35)^. As manifestações clínicas mais comuns são atribuídas ao desconforto respiratório e sobrecarga ventricular direita^(29)^ e são consistentes com o estudo que identificou dispneia, ortopneia, edema, refluxo hepatojugular positivo, dispneia paroxística noturna, congestão pulmonar e aumento da pressão venosa central como CD principais em pessoas com IC descompensada^(21)^. Esses mesmos indicadores ou CDs em pessoas com IC descompensada encontram correlação com o aumento de biomarcadores, como peptídeo natriurético tipo B e peptídeo natriurético pró-cerebral N-terminal, que podem ser utilizados como mais um parâmetro para melhorar a acurácia diagnóstica^(39)^. Ademais, REs da *Nursing Outcomes Classification* (NOC) para o DE VLE também mostraram aplicabilidade clínica de acordo com o aumento de sua pontuação na comparação das médias das avaliações inicial e final em pacientes com IC descompensada^(33)^.

Apesar de exigir precisão que sustentem sua acurácia^(27)^ e de não raro apresentar discordância entre peritos^(30)^, o DE TAD também é um dos mais prevalentes em estudos que identificam DEs em pessoas com quadros de descompensação da IC^(19,37,43)^.

Esse diagnóstico foi o mais identificado em estudo multicêntrico que contrastou os dez DEs mais prevalentes em 302 registros eletrônicos^(23)^, sendo relacionado ao desequilíbrio entre o fornecimento e o consumo de oxigênio^(31)^. Suas principais CDs são relato verbal de fadiga, alteração no eletrocardiograma e resposta anormal da frequência cardíaca à atividade^(29)^.

O FA é um DE isolado da NANDA-I, podendo ser também CD dos DEs DCD, TAD e mais sete DEs ligados a outros domínios e classes^(30)^. Na qualidade de sintoma, fadiga é um fenômeno subjetivo e multifacetado, afetando as dimensões física, psicológica e social^(49)^.

Como DE, revisão integrativa recente identificou FA como um dos mais encontrados^(43)^. Ainda assim, esse DE tem o potencial de ser erroneamente identificado na prática clínica, sendo crucial sua diferenciação de outros DEs, principalmente com TAD, requerendo métodos de avaliação confiáveis para diferenciação^(38)^. Além disso, a necessidade do autorrelato, associada à subjetividade do sintoma, exige treinamento contínuo e específico para o avanço no desempenho de acurácia diagnóstica desse DE^(27)^.

Por fim, a despeito da complexidade que a acurácia diagnóstica impõe, FA constitui DE que demonstra boa evolução dos indicadores de resultados avaliados após a instituição de IE pertinentes^(17)^.

Além da elevada prevalência dos DEs DCD, VLE, TAD e FA, foi possível vislumbrar um comportamento de ocorrência em conjunto para pessoas com IC descompensada. Esse comportamento poderá caracterizar um diagnóstico sindrômico, dado que exibem indicadores e intervenções similares.

### Limitações do estudo

As estratégias de busca adotadas constituem uma limitação intrínseca de revisões integrativas em razão da possibilidade de existência de publicações que não foram contempladas na tática escolhida.

### Contribuições para a área de enfermagem

A atualização sobre os DEs mais frequentemente identificados para pessoas hospitalizadas com IC contribui para o entendimento das principais respostas humanas alteradas em pessoas com IC, direcionando a melhor decisão diagnóstica em busca de resultados em saúde mais assertivos e apoiados em um modelo de prática de enfermagem centrado na pessoa com IC. Por fim, pode subsidiar estudos futuros sobre um possível padrão sindrômico nessa população.

## CONSIDERAÇÕES FINAIS

Este estudo possibilitou identificar que os DEs DCD, VLE, TAD e FA foram os mais frequentemente identificados em pessoas hospitalizadas com IC, agregando evidências que esse conjunto de diagnósticos podem se configurar um diagnóstico de síndrome.

Futuras investigações sobre o agrupamento desses DEs mais frequentemente identificados que ocorrem juntos são necessários para avaliar a existência de um DE de síndrome na NANDA-I em pessoas hospitalizadas com IC.
